# The public health response to a *Plasmodium malariae* outbreak in Penampang district, Sabah during a COVID-19 movement control order

**DOI:** 10.1186/s12936-023-04693-1

**Published:** 2023-10-03

**Authors:** Nurul Athirah Naserrudin, Sam Froze Jiee, Bobby Habil, Anisah Jantim, Ahmad Firdaus Bin Mohamed, Jiloris Julian Frederick Dony, Siti Syarifah Akma Ibrahim, Kimberly M. Fornace, Mohd Rohaizat Hassan, Mohammad Saffree Jeffree, Rozita Hod, Richard Culleton, Kamruddin Ahmed

**Affiliations:** 1 Department of Community Health, Faculty of Medicine, Universiti Kebangsaan Malaysia, Bangi, Malaysia; 2 Sabah State Health Department, Ministry of Health, Kota Kinabalu, Malaysia; 3 Department of Community Medicine and Public Health, Faculty of Medicine and Health Sciences, Universiti Malaysia Sarawak, Kota Samarahan, Malaysia; 4 Penampang District Health Office, Ministry of Health, Kota Kinabalu, Malaysia; 5 Kota Kinabalu Public Health Laboratory, Ministry of Health, Kota Kinabalu, Malaysia; 6 School of Biodiversity, One Health and Veterinary Medicine, University of Glasgow, Glasgow, UK; 7 Faculty of Infectious and Tropical Diseases and Centre for Climate Change and Planetary Health, London School of Hygiene and Tropical Medicine, London, UK; 8 Saw Swee Hock School of Public Health, National University of Singapore and National University Health System, Singapore, Singapore; 9 Department of Public Health Medicine, Faculty of Medicine and Health Sciences, Universiti Malaysia Sabah, Kota Kinabalu, Malaysia; 10 Proeto-Science Center, Ehime University, Matsuyama, Japan; 11 Borneo Medical Health and Research Centre,, Faculty of Medicine and Health Sciences, Universiti Malaysia Sabah, Kota Kinabalu, Malaysia; 12 Department of Pathology and Microbiology, Faculty of Medicine and Health Sciences, Universiti Malaysia Sabah, Kota Kinabalu, Malaysia; 13 Research Center for Global and Local Infectious Diseases, Oita University, Oita, Japan

**Keywords:** *Plasmodium malariae*, Sabah, Malaysia, Malaria outbreak, Healthcare services during COVID-19, Risk-behavior to malaria

## Abstract

**Background:**

Since 2018, no indigenous human malaria cases has been reported in Malaysia. However, during the recent COVID-19 pandemic the World Health Organization is concerned that the pandemic might erode the success of malaria control as there are reports of increase malaria cases in resource limited countries. Little is known how the COVID-19 pandemic has impacted malaria in middle-income countries like Malaysia. Here the public health response to a *Plasmodium malariae* outbreak occurred in a village in Sabah state, Malaysia, during a COVID-19 movement control order is reported.

**Methods:**

An outbreak was declared following the detection of *P. malariae* in July 2020 and active case detection for malaria was performed by collecting blood samples from residents residing within 2 km radius of Moyog village. Vector prevalence and the efficacy of residual insecticides were determined. Health awareness programmes were implemented to prevent future outbreaks. A survey was conducted among villagers to understand risk behaviour and beliefs concerning malaria.

**Results:**

A total of 5254 blood samples collected from 19 villages. Among them, 19 *P. malariae* cases were identified, including the index case, which originated from a man who returned from Indonesia. His return from Indonesia and healthcare facilities visit coincided with the movement control order during COVID-19 pandemic when the healthcare facilities stretched its capacity and only serious cases were given priority. Despite the index case being a returnee from a malaria endemic area presenting with mild fever, no malaria test was performed at local healthcare facilities. All cases were symptomatic and uncomplicated except for a pregnant woman with severe malaria. There were no deaths; all patients recovered following treatment with artemether-lumefantrine combination therapy. *Anopheles balabacensis* and *Anopheles barbirostris* were detected in ponds, puddles and riverbeds. The survey revealed that fishing and hunting during night, and self-treatment for mild symptoms contributed to the outbreak. Despite the index case being a returnee from a malaria-endemic area presenting with mild fever, no malaria test was performed at local healthcare facilities.

**Conclusion:**

The outbreak occurred during a COVID-19 movement control order, which strained healthcare facilities, prioritizing only serious cases. Healthcare workers need to be more aware of the risk of malaria from individuals who return from malaria endemic areas. To achieve malaria elimination and prevention of disease reintroduction, new strategies that include multisectoral agencies and active community participation are essential for a more sustainable malaria control programme.

**Supplementary Information:**

The online version contains supplementary material available at 10.1186/s12936-023-04693-1.

## Background

Sabah is one of the two states of east Malaysia, situated on Borneo Island. In order to provide assistance to traditional sectors and combat poverty, several integrated approaches have been applied in rural areas of the state. Agricultural productivity has driven increases in the standard of living of rural populations and has encouraged farmers to form work-groups or organizations [1]. However, deforestation and the spread of plantations have transformed the ecology and increased human exposure to zoonotic malaria [2–5]. Despite relatively large numbers of *Plasmodium knowlesi* infections in humans, Malaysia has almost eliminated ‘human’ malaria parasite species as the last indigenous human malaria case was reported in 2017 [6], and is currently progressing towards receiving E-2020 malaria elimination certification [7].

Malaysia established a National Malaria Elimination Programme in 1965 [8]. Control measures, such as bed nets, active case detection (ACD), and integrated vector management (IVM), have decreased the prevalence of *Plasmodium falciparum, Plasmodium vivax, Plasmodium ovale,* and *Plasmodium malariae* in Malaysia [7]. However, the World Health Organization (WHO) is concerned that the recent COVID-19 pandemic might erode the success of malaria control, particularly in resource-poor countries [9]. There has been an upsurge in malaria cases and deaths in Cameroon [10] and an increase of 45% malaria cases in Zimbabwe [11]. Little attention has been paid to understanding how the COVID-19 pandemic has impacted malaria in middle-income countries like Malaysia. This report identifies an outbreak initiated by an imported case of *P. malariae* during a governmental movement control order (MCO) as an anti-COVID-19 response. Relatively few studies have focused on the importance of human behaviour in understand the dynamics of malaria transmission during an outbreak.

The aim of the study was to describe the public health response to a *P. malariae* outbreak in a village in Sabah state, Malaysia during a COVID-19 movement control order. It is hoped that the lessons learned by this study may improve case investigation and help to reduce the frequency of future outbreaks during the current pandemic situation.

## Methods

### Outbreak investigation

Moyog sub-district has a land area of 364.68 km^2^ and 25 km from the capital of Sabah, Kota Kinabalu city. The area is surrounded by dense forest. The weather is tropical, with a wet season from November to January. Most of the villagers are farmers. There are also agricultural and plantation based industries. Following the detection of *P. malariae* by real-time polymerase chain reaction (RT-PCR) from a blood sample of a farmer on 16^th^ July 2020 in this district, the Penampang District Health Office declared an outbreak of malaria in a village called Moyog, which is located in Moyog sub-district, Penampang district, Sabah state, Malaysia. Moyog sub-district has a land area of 364.68 km^2^ and 25 km from the capital of Sabah, Kota Kinabalu city. The area is surrounded by dense forest. The weather is tropical, with a wet season from November to January. Most of the villagers are farmers. There are also agricultural and plantation based industries. On 10th July 2020 the blood sample from this primary case was sent to Kota Kinabalu Public Health Laboratory (KKPHL), from the Penampang Primary Health Clinic. According to the Ministry of Health Malaysia regulation, detection of a single introduced or imported human malaria case within a particular locality, is considered an outbreak [8].

Specifically, regarding *P. malariae*, an outbreak is deemed concluded when no additional cases of *P. malariae* are identified within a 120 day period [8]. Consequently, in order to ensure an effective public health response and curb further transmission, Penampang District Health Office organized teams to investigate, control, and manage the outbreak.

ACD was performed by collecting venous blood samples from residents of all villages within 2 km of Moyog village, and thick and thin blood film slides were prepared. This distance was determined based on the known flight range of *Anopheles balabacensis* mosquitoes, which is approximately 500 m [12]. Additionally, it takes into account the distances that communities typically traverse around the villages. The slides were observed by an expert malarial microscopist at Penampang District Health Office. All positive slides were sent to the KKPHL for confirmation of diagnosis by RT-PCR using a commercially available kit (abTES™ Malaria 5 realtime (RT)-PCR II kit, AITbiotech, Singapore).

The index case was defined as the first individual with malaria during the outbreak, and served as the origin of subsequent cases. The primary case refers to an individual who directly acquires the infection through mosquito bites and may contribute to its spread within the outbreak context [13]. Uncomplicated malaria was defined as symptomatic malaria without signs of severe malaria or evidence (clinical or laboratory) of vital organ dysfunction. Severe malaria was defined as presentation with any of the following: coma, metabolic acidosis, severe anaemia, hypoglycaemia, acute renal failure or acute pulmonary oedema [13].

### Location of *P. malariae* cases

A map of cases was created using Open Source Geographic Information System (QGIS). Global Administrative Areas (GADM) (https://gadm.org/data.html) data was used for administrative boundaries. Road data was obtained from OpenStreetMap Data Extracts (https://download.geofabrik.de). Records from the Penampang District Health Office were used for each village's location information included in the district. The coordinates of houses of all cases were determined via GPS.

### Entomological investigation

To assess the vector status and its potential to spread malaria in the affected areas, entomological investigations were carried out by six entomologists and 12 staff from the Entomology Unit of the Sabah State Health Department. They were supported by the Entomology Units from Penampang, and Kota Kinabalu District Health Office. The investigation was conducted from 22nd July to 18th September 2020, three days consecutively from 1800 to 2400 h in every locality. Mosquitoes were caught by the bare leg catch method within 20–50 m radius of the residences of malaria cases. Samples were collected from places where the ecology was conducive to *Anopheles* breeding, such as areas shaded by trees, rock pools near rivers, water-filled animal footprints, and ephemeral puddles [14]. Because *An. balabacensis* is the primary vector of malaria in Sabah [4], the mid-guts and salivary glands of this species were examined under a compound microscope (40 ×) for the presence of *Plasmodium* oocysts and sporozoites, respectively [15]. The parity rate of female mosquitoes was determined by classification based on the WHO malaria terminology as either nulliparous or parous [13]. Mosquito larvae were collected by dipping at potential breeding sites.

### Control measures

To control *P. malariae* transmission further, IVM and health awareness programmes were conducted by Penampang District Health Office officers.

### Malaria beliefs and risk behaviour

To understand beliefs and risk behaviour which might be responsible for malaria transmission, a survey was carried out with adult patients using interview approach by one of the authors (BH) (Additional File 1).

## Results

### Descriptive results

**Fig. 1:**
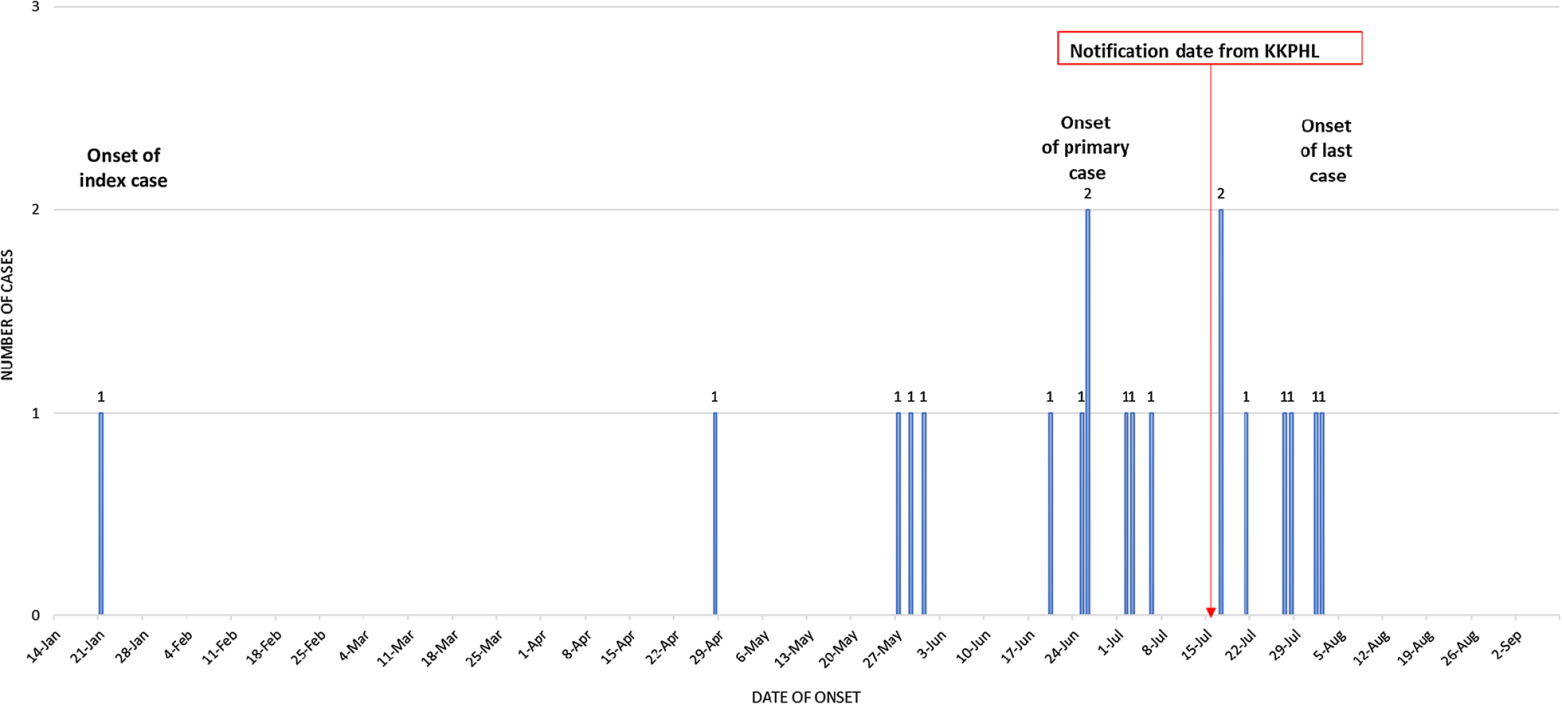
Epidemic curve of the *Plasmodium malariae* outbreak in Moyog sub-district, Penampang district, Sabah, Malaysia from 16th July 2020 until 14th November 2020. The numbers of cases are plotted against the day of onset of fever

From 2010 to 2020, no *P. knowlesi* malaria nor human malaria cases were reported from this district. The outbreak occurred from 16th July 2020 through 14th November 2020 (Fig. 1). Thick and thin blood slides were prepared from samples obtained from all 5254 residents of the following villages (Table 1): Tadong Adong, Mongkusilad, Rugading, Mangkatadan, Notorus, Sosopon, Noungon, Kibunut, Ponombiran, Kuala Kibunut, Pangi, Babagon, Tampasak, Babagon Laut, Timpayasa, Timpangoh Laut, Tirig, Terian, and Moyog. Microscopy identified two villagers with *P. knowlesi* infection, and sixteen villagers with *P. malariae* infection. However, RT-PCR identified all the cases as infected with *P. malariae* only. A total of 18 cases of *P. malariae* were detected from the residents of the following villages: Sosopon, Moyog, Tadong Adong, Mangkatadan, Notorus, Terian, and Babagon Laut. The median parasitaemia level was 1622/µL (ranged 157–10,667/µL) (Table 1). All patients were Malaysian despite 353 smears from samples collected from Indonesian residents. More than 50% of them had been residing in Sabah for more than ten years, while only three of them had been in the village for more than five years. It is not known whether they visited their home country during their residency period.

**Table 1:** The characteristics of patients during the *Plasmodium malariae* outbreak in Moyog sub-district, Penampang district, Sabah, Malaysia

Case ID	Age in years	Gender	Date of onset of fever	Village	Occupation	Parasite count	Gametocyte count
PM001	41	Male	26 June 2020	Moyog	Unemployed	197/µL	
PM002	22	Male	20 June 2020	Sosopon	Assistant engineer	4097/µL	
PM003	9	Male	3 July 2020	Sosopon	Student	157/µL	
PM004	4	Female	28 June 2020	Sosopon	Not applicable	719/µL	
PM005	29	Female	28 June 2020	Sosopon	Housewife	297/µL	
PM006	30	Female	2 June 2020	Tadong Adong	Housewife	10,667/µL	
PM007	35	Male	18 July 2020	Tadong Adong	Unemployed	2234/µL	
PM008	55	Male	21 January 2020	Sosopon	Plantation manager	284/µL	
PM009	19	Male	21 July 2020	Mangkatadan	Contract worker	3480/µL	
PM010	51	Female	28 May 2020	Notorus	Farmer	1208/µL	
PM011	34	Male	30 April 2020	Notorus	Assistant engineer	4020/µL	
PM012	46	Male	2 July 2020	Notorus	Seafarer	1313/µL	
PM013	15	Male	3 August 2020	Terian	Student	1793/$\mu$L	16
PM014	30	Male	18 July 2020	Terian	Farmer	5607/µL	
PM015	48	Male	29 July 2020	Terian	Farmer	1622/µL	
PM016	41	Male	7 July 2020	Babagon Laut	Rubber tapper	315/µL	65
PM017	46	Male	31 May 2020	Babagon Laut	Farmer	9234/µL	1,061
PM018	53	Male	30 July 2020	Babagon Laut	Farmer	1890/µL	31
PM019	55	Male	2 August 2020	Babagon Laut	Farmer	1155/µL	87

PM008, PM001, and PM006 is the index, primary, and severe case, respectively

The *P. malariae* outbreak involved 15 males and four females, making the male: female ratio as 3.7:1. Their median age was 35 (ranged 4–55) years old. There were 16 (84.2%) adults, one adolescent (5.3%) and two children (10.5%). 31.6% of the adult patients were farmers (n = 6), followed by a rubber tapper (n = 1), and a plantation manager (n = 1). The rest of the adults were unemployed (n = 2), an assistant engineer (n = 2), a seafarer (n = 1), a contract worker (n = 1), and housewives (n = 2). The outbreak investigation team identified a Malaysian who worked as a plantation manager in an Indonesian logging company as the index case. He lived in a village called Sosopon, which is also located in the Moyog sub-district.

### The index case of the outbreak

The index case was a 55 year-old male who previously lived and worked in Nabire town in the Papua province of Indonesia. On the 21st January 2020, he had a fever preceded by shivering, which continued for a week. He visited a private clinic, Klinik Gresli, in Naribe. A rapid diagnostic test for malaria was done, which showed a negative result. In early February 2020, he was admitted to a private hospital, MRCC Siloam Hospital, for proper investigation and treatment. He was admitted for 12 days but the patient was not made aware of the diagnosis. Because of his medical condition, he returned to Malaysia on 8th March 2020. He visited the emergency department of Queen Elizabeth Hospital, Kota Kinabalu but since this hospital was designated as a COVID-19 hospital and considered him a non-serious case, he was referred to Penampang Primary Health Clinic for further evaluation and treatment. He made four visits to Penampang Primary Health Clinic for check up on the 14th May, 2nd June, 17th June, and 6th July 2020. He was treated symptomatically without any diagnostic investigation for malaria. He was diagnosed with *P. malariae* by ACD performed in July 2020 after the malaria outbreak was declared. He was diagnosed with *P. malariae* on 25th July 2021 and this was confirmed by RT-PCR.

### The outcome and treatment of *P. malariae* cases

All nineteen *P. malariae* cases were treated with Riamet (artemether-lumefantrine) orally, in the following three tertiary care hospitals in Kota Kinabalu: 15 cases at Queen Elizabeth Hospital, three at Sabah Women and Children’s Hospital, and one at Queen Elizabeth Hospital II. Except one, all cases were uncomplicated malaria. The patient with severe malaria was a 30 year-old pregnant woman who had highest level of parasitemia among all patients (10,667/µL). She has also had acute kidney failure (creatinine 43 mmol/L, urea 2.4 mmol/L, sodium 134 mmol/L and potassium 3.5 mmol/L). Her liver function test also had high protein and albumin levels (albumin 12 g/dL, protein 45 g/dL) with normal liver transaminases profile (alanine transaminases < 6 unit/L, aspartate transaminase 31 unit/L, and alkaline phosphatase145 needs unit/L). All cases recovered after treatment without any adverse events.

### The locations of malaria cases

**Fig. 2:**
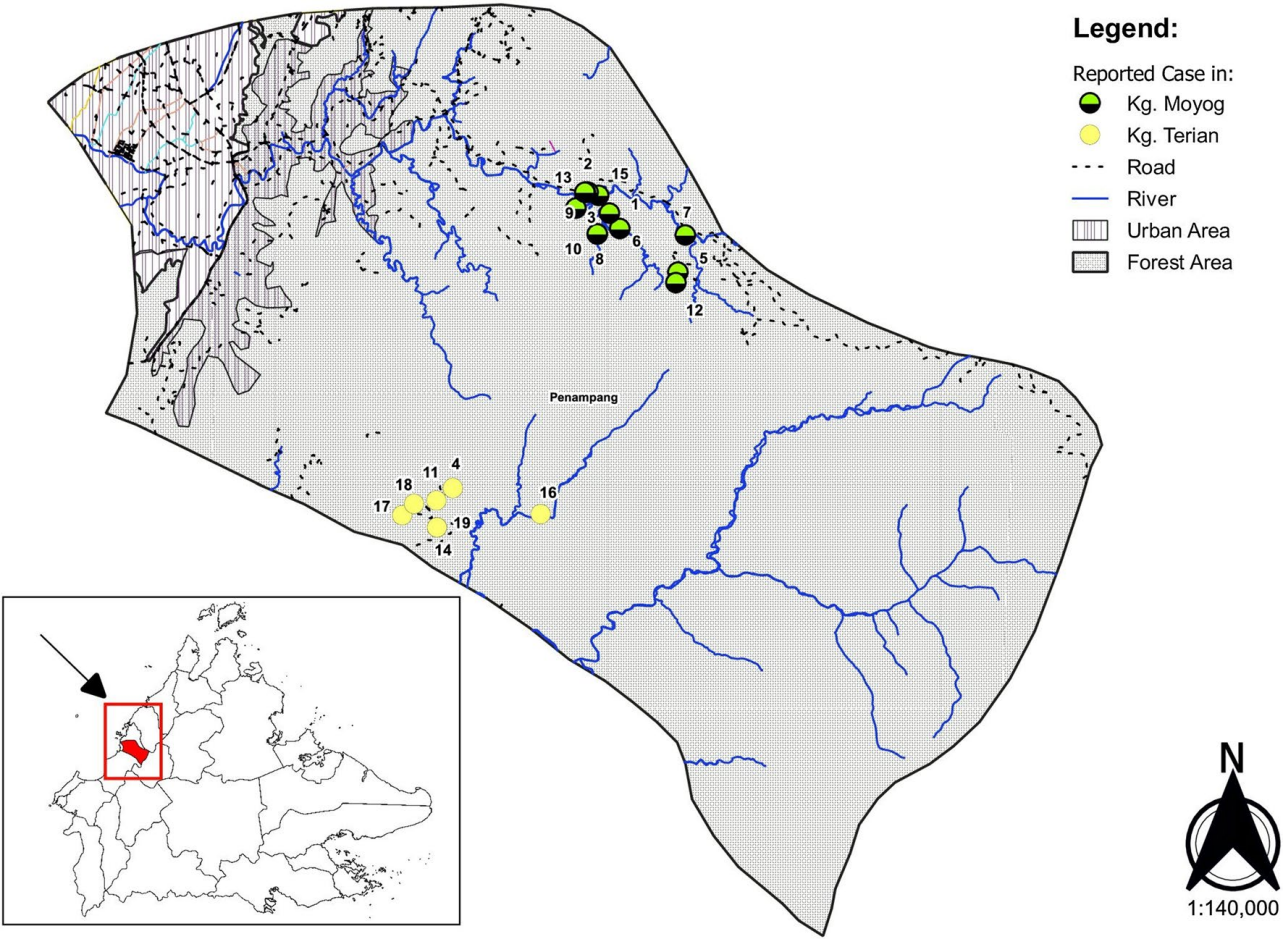
The map of Penampang district showing the residences of the patients with *Plasmodium malariae* positive cases. The lower left inset shows the location of Penampang district in Sabah state. The villages are surrounded by dense forest and far away from urban area. Patients are indicated by green filled circle except the severe case which is indicated by a red filled circle. The number of adjacent to the circle is indicating the serial date of onset of fever, for example the index and last case are 1 and 19, respectively. Fever onset on the same day of patient number 8 and 9, and 13 and 14, The patients are clustered into two due to the location of the villages. The patients interacted in the forest and river for hunting and fishing, respectively. Map was produced using QGIS Version 3.10 (https://www.qgis.org/en/site/forusers/visualchangelog310/index.html). Source of shapefile: https://gadm.org/data.html. GADM shapefile is freely available for academic use

The malaria affected villages are far away from urban areas. They are surrounded by dense forests and there are several nearby rivers. The patients are clustered into two groups due to the closer location of the villages where the patients reside (Fig. 2).

### The entomology studies

A total of 164 adult mosquitoes were captured; 13 of them were female *Anopheles*, 12 were *An. balabacensis* and one *Anopheles tesselatus.* No *Plasmodium* sp. was detected in *An. balabacensis.* In addition, 59% of *An. balabacensis* were parous. A total of 50 *Anopheles* spp. larvae were detected in ponds, puddles, and riverbeds. Among them 24 were *An. balabacensis* and 24 were *Anopheles barbirostris* larvae, but only one larva of each species of *An. tesselatus* and *Anopheles hyrcanus* grp.

### Outbreak control measures

To control *P. malariae* transmission further, IVM such as distribution of bed nets and IRS activities were performed in 779 premises. From 28th August 2020 to 10th October 2020 a health awareness programme was conducted in all 19 villages by local health officers. The health awareness programme included health education, question and answer sessions, preventive measures, and when to seek treatment. Additionally, on 11th October 2020, a workshop was arranged to train 17 villagers to help with future case detection by recognizing early symptoms of malaria and performing blood film for malarial parasite screening.

### Malaria beliefs and risk behaviour

It was found that 74.7% (n = 14/19) of the cases were exposed to malaria due to activities such as fishing and hunting during the night. It was found that except one, 16 of the malaria patients did not use insect repellent (Additional File 2). Only eight used long-sleeved shirts and trousers during outdoor activities, while nine did not. When there was fever nine of them did self-treatment while eight visited a health clinic. None of them used bed nets during sleeping at night. The reasons for not using bed net were as follows: not necessary (7), hot (3), not comfortable (3), no need (3), and did not like (1). Twelve of them were not aware of the activities which might cause them to get malaria, whereas only five of them were aware.

## Discussion

Malaria has not been reported from Penampang district for more than a decade, The outbreak of *P. malariae* described here is, therefore, of significance as the first to occur near Sabah’s capital city Kota Kinabalu, and emphasizes the need to develop more effective public health response strategies for rapid identification, diagnosis, and treatment of cases to prevent the re-introduction or re-establishment of malaria in elimination settings [16]. The study highlights that, despite the travel history of the index case in this outbreak to a malaria endemic region [17], no repeated malaria tests were conducted during healthcare facility visits, which contributed to the *P. malariae* outbreak as described.

Moreover, particularly during events such as the COVID-19 pandemic, the national malaria programme must maintain an ongoing campaign to raise awareness among healthcare workers and communities about the persistent risk of malaria. The public health response must consider regularly updating and training the healthcare workers, as well as ensuring they have adequate resources to prompty address febrile illness, including potential malaria cases. Emphasizing the importance of early diagnosis and treatment for all febrile cases, including presumed malaria, is critical in preventing future outbreaks.

Despite the travel history of the index case in this outbreak to a malaria endemic region [17], the healthcare professionals in Malaysia relied on the negative results of a rapid test for malaria performed in an Indonesian clinic. They did not perform repeat tests for malaria.

*Plasmodium malariae* infection may occur asymptomatically and result in parasitaemia levels near or below detection by light microscopy [18–20]. Polymerase Chain Reaction (PCR) is recognized for its highersensitivity in detecting asymptomatic cases [21, 22]. However, in this outbreak, PCR was not performed on microscopy negative samples. KKPHL is the only place in Sabah to test for malaria by qPCR. At the beginning of the COVID-19 pandemic, it was also the only laboratory to test for COVID-19 cases by RT-PCR, and as a result, its capacity was stretched, negating the possibility of testing all samples for malaria. There remains the possibility that a proportion of these microscopy-negative cases are asymptomatic parasite carriers, constituting an infectious reservoir for the continued transmission of the parasite in the community [19, 20].

The abundance of *An. balabacensis* larvae show that this area is highly suitable for mosquito breeding and so for transmission of malaria. Given the condusive breeding environment and potential for transmission, it becomes imperative to enhance surveillance and case reporting mechanism. This step ensures the prompt detection and reporting of any febrile illnesses, thereby enabling a swift and effective public health response to malaria outbreaks.

For community engagement and education, a group of villagers were trained by the Penampang District Health Office for early response and control of any future malaria outbreak and to integrate them into a future malaria outbreak control team. These approach empowers participants to take proactive measures, for example to perform house-to-house health awareness and education on malaria. To date, no further malaria episodes have been reported from Penampang district. A sensitive and specific molecular amplification method for accurate diagnosis at the point of care is needed to decrease dependence on a centralized diagnostic facility.

**Fig. 3:**
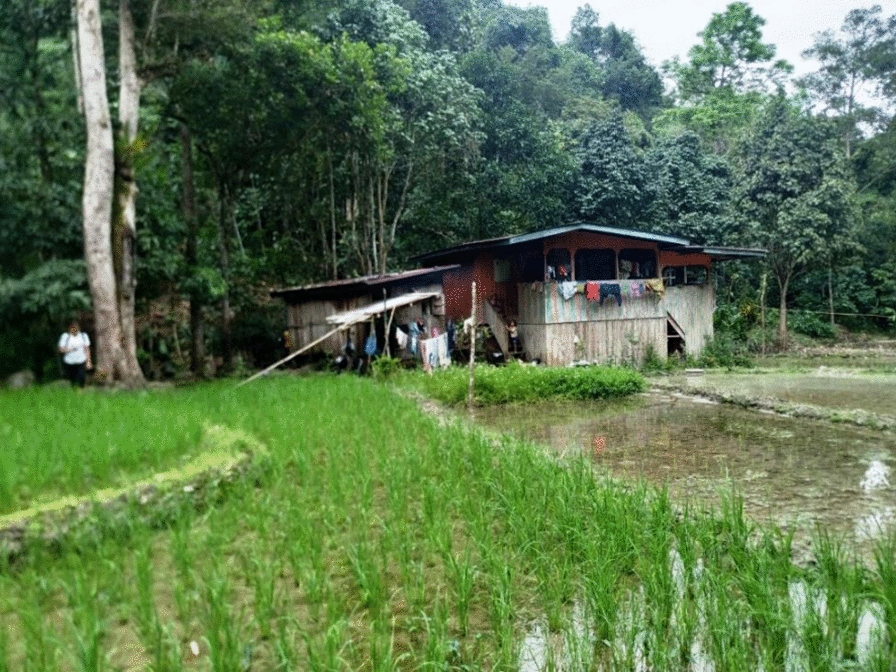
The hut ‘‘sulap’’ near a paddy field in Sosopon village. The paddy field is surrounded by dense forest. Water is accumulated in the field possibly a good environment for mosquito reproduction

In the current outbreak possibly high-risk community behaviour and activities increased vulnerability to malaria infection. Community members commonly engaged in forest-related activities, such as farming, hunting, and spending their time outdoors at night, without taking any effective malaria preventative measures. They did not perceive that outdoor and nighttime activities might expose them to the risk of malaria. Sleeping outdoors in the farm, in a small hut called a “sulap” (Fig. 3) also made them vulnerable to malaria**.** Sulaps are unique wooden structures commonly used by the Kadazan and Dusun ethnicities in Sabah to store their paddy harvest [23].

This survey revealed that when the villagers experienced fever, they prefer to self-treat with antipyretics. This finding is underlined by the fact that all malaria cases identified by ACD had mild symptoms, but they did not seek treatment in healthcare facilities. Furthermore, respondents had no misconceptions regarding malaria transmission but were reluctant to use preventive measures. High-risk behaviour leading to exposure to malaria indicates the need to perform qualitative research to explore the villagers’ understanding of malaria transmission and their belief toward the disease [24–27], which is critical to the success and sustainability of community-based malaria programmes [28]. Anthropological parameters cannot be ignored in malaria control initiatives [25, 28]. Previous studies have shown that misconceptions regarding malaria epidemiology, poor usage of preventive tools for the avoidance of mosquito bites, and the practice of self-treatment put communities at risk of malaria [24, 25].

## Conclusion

In conclusion, it is highly likely that the COVID-19 situation strained health infrastructure and contributed to the outbreak. In situation like this healthcare workers should be vigilant of the risk of other infectious dieseases particularly of malaria in individuals who return from malaria endemic areas. Factors including delays in diagnosis, the disruption of treatment-seeking for febrile illness, self-treatment at home with antipyretics, and the limitation of molecular screening were adversely affected by COVID-19 situation. Along with this, community behaviour, such as nighttime activities and treatment-seeking behaviour contributed to the malaria outbreak. Integrating community education programmes with conventional malaria-preventative measures will be essential for future outbreak management and prevention.

### Supplementary Information

**Additional file 1:** Form used in this study for structured interview.

**Additional file 2:** Malaria beleif and risk behaviour detected among the patients in this outbreak.

## Data Availability

The data supporting the findings of this study are available upon request from the corresponding author.

## Abbreviations

ACD: Active case detection
IVM: Integrated vector management
WHO: World Health Organization
MCO: Movement control order
RT-PCR: Real-time polymerase chain reaction
PCR: Polymerase chain reaction
KKPHL: Kota Kinabalu Public Health Laboratory
QGIS: Open source geographic information system
GDAM: Global administrative areas
IRS: Indoor residual spray
LLIN: Long-lasting insecticidal nets

## References

[1] Ali A, Hassan G, Ngah I, Applanaidu S (2018). Agricultural transformation in Malaysia: the role of smallholders and area development. Agric Transform Incl Growth Inst Agric Food Policy Stud UPM.

[2] Fornace KM, Herman LS, Abidin TR, Chua TH, Daim S, Lorenzo PJ (2018). Exposure and infection to *Plasmodium knowlesi* in case study communities in Northern Sabah, Malaysia and Palawan, the Philippines. PLoS Negl Trop Dis.

[3] Fornace KM, Brock PM, Abidin TR, Grignard L, Herman LS, Chua TH (2019). Environmental risk factors and exposure to the zoonotic malaria parasite *Plasmodium knowlesi* across northern Sabah, Malaysia: a population-based cross-sectional survey. Lancet Planet Health.

[4] Chua TH, Manin BO, Vythilingam I, Fornace K, Drakeley CJ (2019). Effect of different habitat types on abundance and biting times of *Anopheles balabacensis* Baisas (Diptera: Culicidae) in Kudat district of Sabah. Malaysia Parasit Vectors.

[5] Chin AZ, Maluda MCM, Jelip J, Bin JMS, Culleton R, Ahmed K (2020). Malaria elimination in Malaysia and the rising threat of *Plasmodium knowlesi*. J Physiol Anthropol..

[6] Fornace MF, Laporta GZ, Vythilingham I, Chua TH, Ahmed K, Jeyaprakasam NK (2023). Simian malaria: a narrative review on emergence, epidemiology and threat to global malaria elimination. Lancet Inf Dis..

[7] Hussin N, Lim YAL, Goh PP, William T, Jelip J, Mudin RN (2020). Updates on malaria incidence and profile in Malaysia from 2013 to 2017. Malar J.

[8] Vector Borne Disease Sector DCD (2014). Management guideline for Malaria in Malaysia. Infect Dis poverty.

[9] WHO. World Malaria Report (2021). 20 Years of global progress and challenges.

[10] Aborode AT, David KB, Uwishema O, Nathaniel AL, Imisioluwa JO, Onigbinde SB (2021). Fighting Covid-19 at the expense of malaria in Africa: the consequences and policy options. Am J Trop Med Hyg.

[11] Margolin E, Burgers WA, Sturrock ED, Mendelson M, Chapman R, Douglass N (2020). Prospects for SARS-CoV-2 diagnostics, therapeutics and vaccines in Africa. Nat Rev Microbiol.

[12] Verdonschot PFM, Besse-Lototskaya AA (2014). Flight distance of mosquitoes (Culicidae): a metadata analysis to support the management of barrier zones around rewetted and newly constructed wetlands. Limnologica.

[13] WHO (2019). Malaria terminology.

[14] Hawkes FM, Manin BO, Cooper A, Daim S, Homathevi R, Jelip J (2019). Vector compositions change across forested to deforested ecotones in emerging areas of zoonotic malaria transmission in Malaysia. Sci Rep.

[15] WHO (2006). Guidelines for testing mosquito adulticides for indoor residual spraying and treatment of mosquito nets.

[16] Mischlinger J, Rönnberg C, Álvarez-Martínez MJ, Bühler S, Paul M, Schlagenhauf P (2020). Imported malaria in countries where malaria is not endemic: A comparison of semi-immune and nonimmune travelers. Clin Microbiol Rev.

[17] Lubis INDD, Wijaya H, Lubis M (2017). Contribution of *Plasmodium knowlesi* to multispecies human malaria infections in North Sumatera. Indonesia J Infect Dis.

[18] Mueller I, Zimmerman PA, Reeder JC (2007). *Plasmodium malariae* and *Plasmodium ovale* - the “bashful” malaria parasites. Trends Parasitol.

[19] Sutherland CJ (2016). Persistent parasitism: the adaptive biology of malariae and ovale malaria. Trends Parasitol.

[20] Lubis IND, Wijaya H, Lubis M, Lubis CP, Beshir KB, Staedke SG (2020). Recurrence of *Plasmodium*
*malariae* and P falciparum following treatment of uncomplicated malaria in North Sumatera with dihydroartemisinin-piperaquine or artemether-lumefantrine. Open Forum Infect Dis..

[21] Lee PC, Tzyy E, Chong J, Anderios F, Lim YA, Chew CH (2015). Molecular detection of human *Plasmodium* species in Sabah using PlasmoNex™ multiplex PCR and hydrolysis probes rrealtimePCR. Malar J.

[22] Imwong M, Nguyen TN, Tripura R, Peto TJ, Lee SJ, Lwin KM (2015). The epidemiology of subclinical malaria infections in South-East Asia: findings from cross-sectional surveys in Thailand-Myanmar border areas, Cambodia, and Vietnam. Malar J.

[23] Low KO, Lee YF (2012). Investigating the Relationship between Kadazandusun Beliefs about Paddy Spirits, Riddling in Harvest-time and Paddy-Related Sundait. SEA J Gen Stud.

[24] Vilay P, Nonaka D, Senamonty P, Lao M, Iwagami M, Kobayashi J (2019). Malaria prevalence, knowledge, perception, preventive and treatment behavior among military in Champasak and Attapeu provinces, Lao PDR: a mixed methods study. Trop Med Health.

[25] Gryseels C, Uk S, Sluydts V, Durnez L, Phoeuk P, Suon S (2015). Factors influencing the use of topical repellents: implications for the effectiveness of malaria elimination strategies. Sci Rep.

[26] Monroe A, Moore S, Olapeju B, Merritt AP, Okumu F (2021). Unlocking the human factor to increase effectiveness and sustainability of malaria vector control. Malar J.

[27] Finda MF, Moshi IR, Monroe A, Limwagu AJ, Nyoni AP, Swai JK (2019). Linking human behaviours and malaria vector biting risk in south-eastern Tanzania. PLoS ONE.

[28] UNDP/World Bank/WHO Special Programme For Research and Training in Tropical Diseases (2003). The behavioural and social aspects of malaria and its control: an introduction and annotated biliography.

