# Supplementary material for: The public health response to a Plasmodium malariae outbreak in Penampang district, Sabah during a COVID-19 movement control order
Source: Malar J. 2023 Oct 3;22:292. doi: 10.1186/s12936-023-04693-1 (PMC10546630; doi:10.1186/s12936-023-04693-1)
Supplement: Supplementary file 1 — Additional file 1: Form used in this study for structured interview. [file 12936_2023_4693_MOESM1_ESM.docx]

Additional file 1

| **A.** | **Demographic Background** | |
| --- | --- | --- |
|  | Name |  |
|  | Age |  |
|  | Occupation |  |
|  | Race |  |
|  | Household number |  |
|  | Education level  (√) please tick | ( ) No formal education  ( ) Primary school  ( ) Secondary school  ( ) Tertiary education (college/university) |
| **B** | **Knowledge** | |
| i. | Signs and symptoms of malaria | ( ) Fever  ( ) Loss consciousness  ( ) Vomit  ( ) I don’t know |
| ii. | Can malaria be transmitted from a person to another person? | ( ) Yes  ( ) No  ( ) I don’t know |
| iii. | How does malaria spread? | ( ) Mosquito bite  ( ) Touching a malaria patient  ( ) Food/Drink  ( ) I don’t know  ( ) Others :: |
| iv. | How to detect malaria? | ( ) Blood screening  ( ) Eye-check  ( ) I don’t know  ( ) Others : : |
| v. | What are the effects of malaria? | ( ) Coma  ( ) Liver failure  ( ) Kidney failure  ( ) Anemia  ( ) Lymphadenitis  ( ) Miscarriage  ( ) Other:  ( ) I don’t know |
| vi. | How to prevent malaria? | ( ) Sleep on insecticide treated bed nets  ( ) Insect repellent  ( ) Others  ( ) I don’t know |
| **C** | **Attitude** | |
| i. | Malaria is a dangerous disease | ( ) Yes  ( ) No |
| ii. | Avoiding mosquito bites can prevent malaria | ( ) Yes  ( ) No |
| iii. | It is necessary to take medicine to prevent malaria even if a person has no symptoms and signs | ( ) Yes  ( ) No |
| iv. | I am willing to have a blood test even if not sick | ( ) Yes  ( ) No |
| v | The community should be involved in activities to prevent malaria | ( ) Yes  ( ) No |
| **D.** | **Practise** | |
| i. | Have you ever been given information about malaria | ( ) Yes  ( ) No |
| ii. | What would you do if you had a fever? | ( ) Go to the clinic/hospital  ( ) Home treatment  ( ) Traditional healer  ( ) Others: |
| iii. | Do you use a mosquito bed net while sleeping at night? | ( ) Yes  ( ) No |
| iv. | If you do not use a mosquito net, please state why it is not used | Please explain |
| v. | Do you often go out at night/ dawn | ( ) Yes  ( ) No |
| vi | If you answer yes for question D(vi), please state the time | Please state the time here : |
| vii. | If you answer yes for question D(v), please describe your activities | ( ) Hunting  ( ) Fishing  ( ) Rubber tapping  ( ) Others |
| viii | Did you know that activities at night can expose you to mosquito bites? | ( ) Yes  ( ) No  ( ) Others |
| ix | Do you wear long -sleeved shirts and long pants when doing outdoor activities to avoid mosquito bites? | ( ) Yes  ( ) No |
| x | Do you use repellents when doing outdoor activities to prevent mosquito bites | ( ) Yes  ( ) No |
| xi | What do you do to avoid mosquito bites | ( ) Wearing insecticide treated bed nets  ( ) Insect repellent  ( ) I don’t know  ( ) Others : |
| xii | The best/preferred way to get information about Malaria | ( ) Reading poster or pamphlet  ( ) Health talk  ( ) Individual advice  ( ) Others |
