# Supplementary material for: The public health response to a Plasmodium malariae outbreak in Penampang district, Sabah during a COVID-19 movement control order
Source: Malar J. 2023 Oct 3;22:292. doi: 10.1186/s12936-023-04693-1 (PMC10546630; doi:10.1186/s12936-023-04693-1)
Supplement: Supplementary file 2 — Additional file 2: Malaria beleif and risk behaviour detected among the patients in this outbreak. [file 12936_2023_4693_MOESM2_ESM.docx]

Additional file 2

|  | Yes | No | Children were excluded from the interview |
| --- | --- | --- | --- |
| Use insect repellant during outdoor activities | 1 | 16 | Not applicable |
| Wear long sleeved shirt and trouser during outdoor activities | 8 | 9 | Not applicable |
| Do self-treatment during fever | 9 | 8 | Not applicable |
| Use bed net during sleep at night | 0 | 17 | Not applicable |
| Aware of activities that may cause getting malaria | 5 | 12 | Not applicable |
